# NF-κβ: A Potential Target in the Management of Vascular Complications of Diabetes

**DOI:** 10.3389/fphar.2017.00798

**Published:** 2017-11-07

**Authors:** Sachin V. Suryavanshi, Yogesh A. Kulkarni

**Affiliations:** Shobhaben Pratapbhai Patel School of Pharmacy and Technology Management, SVKM’s Narsee Monjee Institute of Management Studies, Mumbai, India

**Keywords:** NF-κβ, diabetic complications, inflammation, nephropathy, neuropathy, retinopathy, cardiomyopathy, NF-κβ inhibitors

## Abstract

Diabetes is a metabolic disorder affecting large percentage of population worldwide. NF-κβ plays key role in pathogenesis of vascular complications of diabetes. Persistent hyperglycemia activates NF-κβ that triggers expression of various cytokines, chemokines and cell adhesion molecules. Over-expression of TNF-α, interleukins, TGF-β, Bcl2 and other pro-inflammatory proteins and pro-apoptotic genes by NF-κβ is key risk factor in vascular dysfunction. NF-κβ over-expression also triggers calcification of endothelial cells leading to endothelial dysfunction and further vascular complications. Inhibition of NF-κβ pro-inflammatory pathway is upcoming novel target for management of vascular complications of diabetes. Various natural and synthetic inhibitors of NF-κβ have been studied in management of diabetic complications. Recent preclinical and clinical studies validate NF-κβ as promising target in the management of vascular complications of diabetes.

## Introduction

Diabetes mellitus (DM) and its associated complications are one of the major leading causes of mortality in public worldwide (IDF, 2015; WHO, 2016). The burden of diabetes has increased in India from 5.8 to 8.7% since year 2000 to 2015 (Unnikrishnan et al., 2016; Pradeepa and Mohan, 2017). Chronic hyperglycemic condition has devastating effects on vasculature that ends into different micro-vascular and macro-vascular complications like neuropathy, nephropathy, retinopathy and cardiomyopathy (He and King, 2004). Increased levels of advanced glycation end products (AGEs), receptors for it (RAGE), oxidative stress, lipoproteins and hyperlipidemia enhance expression of nuclear factor-κβ (NF-κβ) by various pathways. Furthermore, inappropriately expressed NF-κβ augments apoptosis and inflammatory process that plays principle role in cell injury and further complications (Singh et al., 2014).

The NF-κβ is a DNA binding protein factor which is involved in transcription of different pro-inflammatory and inflammatory molecules like cytokines, chemokines, cell adhesion molecules (CAM) and different enzymes (He and King, 2004). The expression of cytokines and inflammatory molecules plays an important role in pathophysiology of diabetes and its associated micro-vascular and macro-vascular complications via modulating different NF-κβ pathways (Patel and Santani, 2009).

The current review focuses on role of NF-κβ in pathophysiology of various vascular complications of diabetes and effect of NF-κβ inhibitors in the management of same.

## NF-κβ Family

NF-κβ is an evolutionarily conserved protein form the Rel family found in all cell types (Lawrence, 2009). Rel family/NF-κβ regulates the expression of numerous genes involved in control of various normal cellular and sub-cellular processes like inflammatory and immune responses, cellular growth, cell development and cell survival (Gilmore, 2006; Perkins, 2007). NF-κβ is also involved in control of responses to numerous stimuli such as free radicals, stress, cytokines, ultraviolet radiations and viral and bacterial antigens (De Martin et al., 2000; Lawrence, 2009).

The NF-κβ family includes five related transcription factors: p50 (NF-κβ1), p52 (NF-κβ2), p65 (RelA), c-Rel, and RelB (Hayden and Ghosh, 2011). NF-κβ1 and NF-κβ2 are produced from processing of precursors p105 and p100, respectively (Ghosh and Karin, 2002; Gilmore, 2006; Patel and Santani, 2009). These transcription proteins possess dimerization domain to which DNA binds; Rel homology domain (RHD) at N-terminal facilitates site for dimerization through which they forms homo or hetero–dimers (Ghosh and Karin, 2002; Hayden and Ghosh, 2011). The most copious forms of dimers include p65/p65 homodimers and p65/p50 heterodimers (Birbach et al., 2002). RelA, RelB, and c-Rel contains *trans*-activation domains (TADs) at C-terminal that allows binding of DNA and activation of target gene expression. Despite this, p50/p105 and p52/p100 do not contains TAD, they participate in target gene expression by forming heterodimers with RelA, RelB or c-Rel (Li and Verma, 2002; Hayden and Ghosh, 2004). Instead of TAD, p50/p105 and p52/p100 contains long ankyrin repeat–containing domain (ARD). The NF-κβ1 and NF-κβ2 contains 6–7 ankyrin repeats containing 33 amino acid sequences that facilitate site for dimerization (Hayden and Ghosh, 2011; Napetschnig and Wu, 2013).

## Activation of NF-κβ Family

The activation of NF-κβ is regulated by family of inhibitors of NF-κβ (IκB). The IκB family contains various regulatory proteins (IκBα, IκBβ, IκBγ, and Bcl-3) that keep NF-κβ inactive in cytoplasm (Gilmore, 2006; Baker et al., 2011; Hayden and Ghosh, 2011). Another participant in NF-κβ pathway is IκB kinase (IKK) complex which catalyzes IκB to release NF-κβ (Perkins, 2007). The IKK complex consists of two catalytically active kinases, IKKα (IKK1) and IKKβ (IKK2) and a regulatory scaffolding protein NF-κβ essential modifier (NEMO) that keep NF-κβ inactive in the cytoplasm (Zheng et al., 2010; Liu and Chen, 2011).

The NF-κβ is activated via two pathways *viz*. canonical pathway and non-canonical pathway (Zheng et al., 2010; Baker et al., 2011). In canonical pathway when signal is transduced, NEMO-containing IKK complexes are activated and induce phosphorylation of IKK complex via ubiquitination leading to release of NF-κβ dimers (Karin and Ben-Neriah, 2000). Furthermore the NF-κβ dimers enter in the nucleus and modulate target gene expression. In non-canonical pathway, NF-kβ inducing kinase (NIK) enhance phosphorylation of IKKα and IKKβ, NEMO independently to release NF-κβ dimers (Hayden and Ghosh, 2011; Liu and Chen, 2011; Shih et al., 2011).

## Role of NF-κβ in Pathogenesis of Vascular Complications

Prolonged hyperglycemia and insulin resistance are key players in diabetic vascular complications (Ruderman et al., 1992). Hyperglycemia induces formation of AGEs and overproduction of reactive oxygen species (ROS) (Paneni et al., 2013; Tobon-Velasco et al., 2014). The key pathways involved in production of AGEs and ROS due to hyperglycemia includes increased polyol flux, activation of protein kinase – C (PKC), increased intracellular formation of AGEs and increased hexosamine flux (Brownlee, 2001, 2005; Xing et al., 2016). Consecutively, ROS and AGEs initiate pro-inflammatory response and endothelial dysfunction via activation of NF-κβ (Paneni et al., 2013; Xing et al., 2016). AGEs bind to RAGE present on cell surface of vascular smooth muscles and directly activate NF-κβ (Lander et al., 1997).

Evidence suggests that activation of NF-κβ is essential for cell proliferation and cell migration (Bellas et al., 1995). In hyperglycemic condition the NF-κβ activity is enhanced significantly leading to release of cytokines, TGF-β, chemokines and vesicular cell adhesion molecules (VCAMs) (Patel and Santani, 2009). Consequently, up-regulation of TNF-α, IL1β, IL6, CD36, MCP-1 leads to endothelial cell apoptosis and inflammatory process (Reddy and Natarajan, 2011; Evans and Goldfine, 2016). Additionally, over-activated NF-κβ carry out abnormal transcription of DNA and various genes involved in vascular complications (Zheng et al., 2010). Over-activity of NF-κβ also leads to altered gene expression of vascular endothelial growth factor (VEGF), platelet-derived growth factor (PDGF), endothelin-1 (ET-1), activated protein C (APC) and transforming growth factor-β (TGF-β) that ends in to vascular cell damage and angiogenesis (Kitada et al., 2010).

Increased TNF- α, IL-6 and IL-10 have been observed in adipose tissues in obese rats (de Luca and Olefsky, 2008). TNF-α, and cytokines activates NF-κβ and recruit monocytes producing macrophages M1 and M2 that promote β-cell destruction and insulin resistance. This is key factor in pathophysiology of atherosclerosis (Baker et al., 2011). AGE/RAGE increases vascular calcification through activation NF-κβ activation and increased expression of TGF-β, mitogen activated protein kinase (MAPK), and PKC leading to hardening of medial layer of blood vessels (Kay et al., 2016).

## NF-κβ and Diabetic Nephropathy

Diabetic nephropathy (DN) is the leading cause of cardiovascular mortality and chronic kidney disorder (CKD) in diabetic patients. DN is characterized by persistent microalbuminuria, decreased glomerular filtration (GFR) rate and increased albumin to creatinine ratio (Reutens and Atkins, 2011). Glomerular changes such as thickening of capillary basement membrane, mesangial expansion and glomerulosclerosis are pathological indications of DN (Patel and Santani, 2009; Lopez-parra et al., 2012). The recent reports endorse about 12–55% incidences of end stage renal disorders (ESRD) are attributed to diabetes (WHO, 2016). The prevalence of microalbuminuria among diabetic patient in India was found to be 24.3% (Pradeepa and Mohan, 2017).

Nevertheless, intra-renal inflammation is key factor in pathophysiology of DN. Accumulation of macrophages, monocytes, T-cells, and fibroblasts in diabetic kidney are responsible for inflammation in DN (Bohle et al., 1991; Sakai et al., 2005; Sanz et al., 2010). The activation of NF-κβ pathways by TNF-α and other cytokines are responsible for accumulation of macrophages in human DN (Sakai et al., 2005; Lenz et al., 2008). There are two sub types of macrophages; M1 and M2. M1 macrophages are involved in pro-inflammatory response while M2 macrophages are involved in tissue repair remodeling and angiogenesis process (Schmid et al., 2006; Silva et al., 2011; Lopez-parra et al., 2012). In diabetic patient, increased AGEs and ROS burden activates leukocytes that release superoxide radicals and proteases in the kidney (Mohamed et al., 1999; Patel and Santani, 2009). Additionally, leukocytes up regulate transcription of NF-κβ in endothelial and mesangial cells (Sanz et al., 2010; Silva et al., 2011; Borgohain et al., 2017).

**FIGURE 1:**
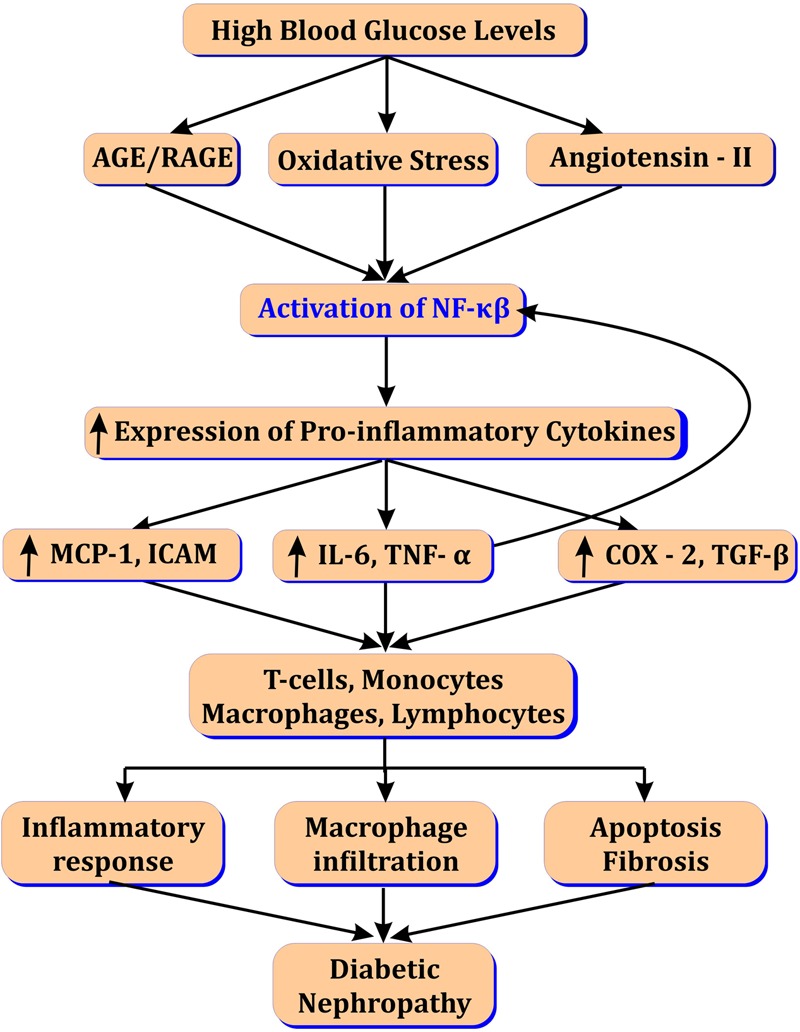
Role of NF-κβ in diabetic nephropathy.

Activation of NF-κβ up-regulate monocyte chemoattractant protein-1 (MCP-1) leading to macrophage infiltration (Cha et al., 2005), renal injury and increased microalbuminuria in DN (Lopez-parra et al., 2012). NF-κβ also enhances expression of TGF-β-activated kinase (TAK-1) from MAPK family known as MAP3K7. In turn TAK1 induce activation of transforming growth factor (TGF-β) leading to extracellular matrix accumulation and fibrosis in the diabetic kidney (Choi et al., 2012; Kanasaki et al., 2013; Meng et al., 2015). MAPK also contribute in gene over-expression of various cytokines and intracellular adhesion molecules (ICAM), c-Jun NH_2_-terminal kinase (JNK) and leukocyte infiltration via NF-κβ activation (Sakai et al., 2005; Pan et al., 2013). Renal podocyte injury and podocyte protein accumulation is hallmark of DN. Angiotensin II levels are increased in response to elevated AGEs and oxidized lipids in DN (**Figure 1**). This in turn, activates NF-κβ via angiotensin (AT_1_ and AT_2_) receptors and activation of transient receptor potential canonical (TRPC) (Ilatovskaya et al., 2015). NF-κβ increase calcium influx and ROS canonically in diabetic kidney leading to podocyte protein accumulation and injury (Lee, 2004; Campbell et al., 2011).

## NF-κβ Inhibitors in Diabetic Nephropathy

Inhibition of NF-κβ activation may provide treatment option in DN by inhibiting transcription of genes and blocking inflammatory process. A few researchers studied the effect of NF-κβ inhibition on DN. Curcumin is the active polyphenol component of herbal medicine *Curcuma longa* well known as turmeric. Curcumin treatment improved DN in type I diabetic rats. It inhibited macrophage infiltration via NF-κβ inhibition. It also inhibited degradation of NF-κβ regulatory protein IκBα leading to decreased expression of pro-inflammatory (TNF-α, IL-1β) and profibrotic cytokines (ICAM-1, MCP-1, and TGF-β_1_) (Soetikno et al., 2011).

Pal and co-workers studied the effect of mangiferin in rats with DN. Mangiferin; a natural C-glucosyl xanthone and polyphenol obtained from bark of *Mangifera indica* (Mango tree) has antioxidant activity, thereby it inhibited AGEs and oxidative stress mediated pro-inflammatory signaling cascade. Mangiferin inhibited oxidative stress via inhibition of PKC, MAPK and TGF-β and improved fibrosis in diabetic kidney. It also reduced expression of pro-apoptotic proteins Bcl-3 and caspase-9 via inhibition of NF-κβ and TNF-α. Mangiferin also decreased expression of NF-κβ, IKKα and inhibited degradation of IκBα (Pal et al., 2014).

Borgohain and co-workers studied effect of naturally occurring piceatannol on renal inflammation in alloxon-induced DN in rats. Piceatannol is analog of resveratrol and small molecule present in plant *Euphorbia lagascae*. Piceatannol decreased superoxide dismutase (SOD) and glutathione (GSH) and increased malondialdehyde (MDA) and nitric oxide (NO) levels in kidney restoring oxidative stress. Moreover, treatment with piceatannol inhibited NF-kB p65/p50 binding to DNA and reduced renal pro-inflammatory cytokines like TNF-α, IL-1β and IL-6 (Borgohain et al., 2017).

Xu and co-workers studied the effect of resveratrol on renal inflammation and mesangial cell proliferation in streptozotocin induced diabetic mouse. Resveratrol showed renoprotective action via decreasing activation of NF-κβ and inhibition of Akt and JNK. Moreover resveratrol also inhibited NF-κβ dependent activation of plasminogen activator inhibitor (PAI-1) and ICAM leading to blockade of pro-apoptotic cascade (Xu et al., 2014).

Liu and co-workers studied the effect of berberine on NF-κβ pathway in alloxan induced diabetic renal injury in mice. Berberine is an isoquinoline alkaloid isolated from *Cortex Phellodendri* and *Coptidis rhizoma*. It has hypolipidemic, antihyperglycemic and antioxidant activity (Singh and Kakkar, 2009). Berberine showed improvement in diabetic renal injury through inhibition of NF-κβ and thereby down-regulation of ICAM, TGF-β1 and fibronectin. It also restored IκBα levels by inhibiting its degradation in kidney tissue. As a result, berberine reduced accumulation of extracellular matrix in kidney cells (Liu et al., 2010).

NF-κβ activation and macrophage infiltration in adipose tissue has been concerned as key mechanism in development of insulin resistance in diabetic patients (Zamboni et al., 2007). Celastrol, a pentacylic triterpenoid compound isolated from the roots of *Celastrus regelii* and *Tripterygium wilfordii* has been studied for its effect on insulin resistance and renal injury in db/db mice. Celastrol showed improvement in insulin resistance and renal injury via inhibition of NF-κβ pathways and inhibiting expression of inflammatory mediators like IFNγ, NOX4, TLR4, and TNF-α (Kim et al., 2013).

Jianfang and co-workers studied the effect of Paeoniflorin, a natural product obtained from plant *Paeonia lactiflora* in DN in rats. Paeoniflorin showed improvement in DN by suppressing expression of iCAM-1 and Collagen – IV via inhibition of NF-κβ. It also reduced macrophage infiltration and renal hypertrophy (Jianfang et al., 2009).

Recent studies revealed that (2E,6E)-2,6-bis (2-(trifluoromethyl) benzylide-ne)cyclohexanone (C66), a synthesized curcumin analog inhibited JNK2 protein form MAPK family and thereby inhibited NF-κβ activity and showed reno-protective action by blocking pro-inflammatory cytokines expression (Pan et al., 2013). 1,25-Dihydroxyvitamin D3, a hormonal form of vitamin D have negative effect on rennin angiotensin system (RAS) and regulate calcium influx in the kidney (Li et al., 2002). 1,25-Dihydroxyvitamin D3 prevented renal injury via inhibition of RAS system and NF-κβ induced pro-inflammatory cascade (Zhang et al., 2007).

Fenofibrate, an antihyperlipidemic drug and peroxisome proliferator-activated receptor alpha (PPARα) activator was investigated for anti-inflammatory response through NF-κβ inhibition in DN rats. Fenofibrate treatment reduced expression of NF-κβ p65, PAI-1, and ICAM-1 along with remarkable improvement in lipid profile in rats through activation of PPARα. Fenofibrate provided renoprotective action via inhibition of NF-κβ pro-inflammatory pathways (Chen et al., 2008). Thiazolidinedione a PPARα activator also showed protective effect in renal injury through anti-inflammatory effects mediated by inhibition of NF-κβ activation in experimental diabetic rats (Ohga et al., 2007). Cerivastatin, a synthetic HMG-CoA reductase inhibitor has been studied for its protective effect in DN in rats. Cerivastatin showed renoprotective action through inhibition of NF-κβ, ICAM and macrophage infiltration (Usui, 2003).

Curcumin has been proved clinically for its beneficial effects in DN via inhibition of NF-κβ in randomized double blind and placebo controlled clinical trial. Oral administration of curcumin attenuated expression of TGF-β, IL-8 and proteinuria in type-2 diabetic patients with nephropathy (Khajehdehi et al., 2011; Lv et al., 2015; Prabhakar, 2017). Administration of alpha lipoic acid in DN patients reduces oxidative stress via inhibition of NF-κβ and inflammatory cytokines such as TNF-α and IL-8 (Lv et al., 2015).

## NF-κβ and Diabetic Neuropathy

Diabetic neuropathy is the most common and stubborn vascular complication of diabetes and major cause of mortality (Boulton et al., 2005). It involves the sensory loss or dysfunction of autonomic, peripheral, somatic sensory and motor nerves (Aslam et al., 2014). Distal polyneuroathy (DPN) and autonomic neuropathy are the most common amongst various types of diabetic neuropathies. Pathological changes include loss of nerve fibers, axonal thickening, demyelination of nerves and neuronal capillary narrowing (Thomas, 1999; Aslam et al., 2014). The prevalence of diabetic neuropathy is higher as compared to other complications (Yagihashi et al., 2011).

The patients with uncontrolled high blood sugar levels experience uncomfortable sensory symptoms especially in lower limbs. The vibration perception threshold and nerve conduction velocity is drastically reduced in diabetic neuropathy (Thomas, 1999; Yagihashi et al., 2007). High blood sugar levels triggers the production of oxidative stress and AGE/RAGE formation in neuronal cells (Vincent et al., 2002; Xing et al., 2016). Increased glycated hemoglobin (HbAc_1_), and stromal collagen level in peripheral nerves, schwann cells and endoneurial vessels is another risk factor for progression of peripheral nerve injury (Sugimoto et al., 1997). Increased AGE/RAGE, ROS, and HbAc_1_ in nerve fibers activates apoptosis and insulin resistance via activation of NF-κβ and release of TNF-α (Haslbeck et al., 2005; Yagihashi et al., 2007, 2011).

**FIGURE 2:**
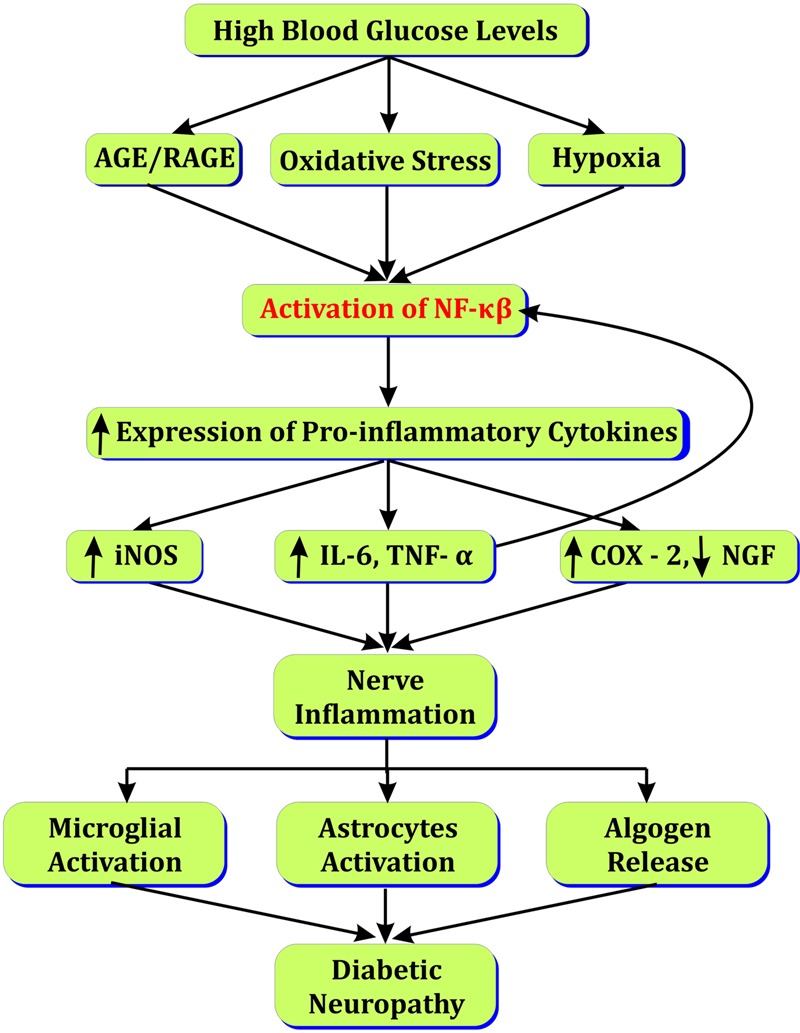
Role of NF-κβ in diabetic neuropathy.

Increased polyol flux by aldose reductase contributes in accumulation of sorbitol and generation of ROS in neuronal cells (Brownlee, 2005). Sorbitol and ROS in nerves affects Na^+^,K^+^ ATPase activity that delays nerve conduction velocity (Zhao et al., 2011). NF-κβ over-expression due to increased ROS, PKC, and AGEs leads to leukocyte infiltration and decreased neuronal growth factor (NGF), IL6, IL1β, and TNF-α in nerve cell (Okamoto et al., 2001; Vincent et al., 2002; Pittenger et al., 2003). Arachidonic acid pathway is activated in response to NF-κβ activation that increases COX-2 concentration nerve cells (Yagihashi et al., 2011). Increased oxidative stress activates stress kinase MAPK leading to nerve injury (Tomlinson, 1999). Increased ICAM and NF-κβ expressions are observed in microvessels of sciatic–tibial nerves of diabetic rats leading to narrowing of vessels and ischemic conditions following inflammatory response (Wang et al., 2006). Peroxisome proliferator-activated receptors (PPARs) are reduced in nerves in response to increased chemokines that enhance gene expression and neuronal death (**Figure 2**; Freitag and Miller, 2014).

## NF-κβ Inhibitors in Diabetic Neuropathy

Various natural and synthetic NF-κβ inhibitors have been studied for their protective effect in diabetic neuropathy. Curcumin was studied for its analgesic activity in diabetic neuropathy in mouse (Sharma et al., 2006) and rat (Li et al., 2013). Curcumin inhibited inducible nitric oxide synthase (iNOS) levels and serum TNF-α and TNF-α receptor 1 in nerve fiber by inhibiting NF-κβ expression. Curcumin also reduced neuropoietic cytokines such as IL-1, IL-6 (Joshi et al., 2013).

Another natural compound resveratrol provide neuroprotective and anti-inflammatory activity through inhibition of NF-κβ activation (Kumar and Sharma, 2010). Resveratrol also inhibited TNF-α, COX-2, and IL-6 levels contributing to NF-κβ activation. Additionally resveratrol also inhibited degradation of IκB-α protein.

The role of NF-κβ and erythroid 2-related factor 2 (Nrf2) has been explored in diabetic neuropathy (Li et al., 2008; Ganesh Yerra et al., 2013; Wardyn et al., 2015). It is clear that Nrf2 up-regulation is linked with the NF-κβ inhibition. Sulforaphane, a natural isothiocyanate present in *Brassica oleracea* (broccoli) has been studied in diabetic neuropathy. Sulforaphane is a potent inducer of Nrf2 and inhibitor of NF-κβ. Sulforaphane inhibited mechanical hyperalgesia. It also inhibited IKKβ phosphorylation, IL-6 and TNF-α levels in sciatic nerve indicating inhibition of NF-κβ activation. From above study it is clear that NF-κβ inhibition by sulforaphane provides protective effect in diabetic neuropathy (Negi et al., 2011a).

The traditional herbal medicine *Acorus calamus*, has been studied for its antihyperglycemic activity, insulin sensitizing activity (Wu et al., 2009) and neuroprotective effect (Muthuraman and Singh, 2011). Alcoholic extract of *A. calamus* showed anti-diabetic activity by suppressing Glucose-6-phosphatase and Fructose-1,6-bisphosphatase enzyme activities (Prisilla et al., 2012). The ethyl acetate fraction of AC showed insulin sensitizing activity by α-glucosidase inhibition and PPAR-γ agonist activity (Wu et al., 2009). The hydroalcoholic extract of AC attenuate neuropathic pain via its anti-inflammatory property (Muthuraman and Singh, 2011). AC possess anti-inflammatory activity probably via inhibition of NF-κβ activation (Kim et al., 2009). From literature it can be said that *A. calamus* may provide neuroprotection, and analgesic activity in diabetic neuropathy via inhibition of NF-κβ.

Pioglitazone, a thiazolidinedione derivative and a PPAR-γ agonist have been studied in diabetic neuropathy. Pioglitazone inhibited the PKC pathway by activating PPAR-γ receptors and improved peripheral nerve function. Pioglitazone also inhibited NF-κβ activation and MAPK levels in the peripheral nerves and provided anti-inflammatory activity (Yamagishi et al., 2007). Pregabalin, a nutraceutical and first drug approved by FDA for treatment of diabetic neuropathy is a potent NF-κβ inhibitor. Pregabalin inhibited NF-κβ activation through nuclear localization of p65 in nerve cells. It also inhibited NF-κβ regulated cytokine and chemokines such as COX-2, TNF-α, and IL-6 (Verma et al., 2014).

Melatonin also modulates neuro-inflammation through activation of Nrf2, inhibition of NF-κβ activation and degradation of IκBα. Treatment with melatonin reduced pro-inflammatory cytokines such as TNF-α and IL-6. The COX-2 and iNOS levels were reduced in nerve fibers. The inhibition of NF-κβ by melatonin reduced DNA fragmentation and improved diabetic neuropathy. The melatonin reduced ROS mediated inflammatory mediators like TNF-α, IL-6, and COX-2; thereby DNA fragmentation. Melatonin reduced expression of iNOS and degradation of IκBα. Furthermore, melatonin also inhibited NF-κβ by increasing Nrf2 and heme oxygenase-1 (HO-1) levels in sciatic nerves diabetic rats (Negi et al., 2011b).

Antioxidants such as alpha lipoic acid reduced oxidative stress and showed anti-inflammatory activity via inhibition of NF-κβ (Jeong et al., 2011). Alpha-lipoic acid showed promising results in patients with diabetic neuropathy via inhibition of NF-κβ. Alpha-lipoic acid dose dependently inhibit expression of NF-κβ and thereby down regulate expression of iCAM and VCAM (Vallianou et al., 2009; Sandireddy et al., 2014).

## NF-κβ and Diabetic Retinopathy

Diabetic retinopathy (DR) is one of the most specific micro-vascular complication and primary cause of blindness in diabetic patients (WHO, 2016). The etiology of DR includes loss of pericytes, capillary basement thickening, microaneurysm, cataract, capillary acellularity, and breakdown of blood–retina barrier (Zhang et al., 2012). Depending on severity, DR is generally classified into proliferative DR (PDR), Non-proliferative DR (NPDR), and diabetic macular edema (DME) (Wu, 2013).

Recent reports state that in 2010, one third of an estimated 285 million people with diabetes have signs of DR (Yau et al., 2012; Lee et al., 2015). It is estimated that the number of people with DR will rise up to 191.0 million by 2030 (IDF, 2015). The prevalence of DR and vision-threatening DR (VTDR) in United States during 2005 to 2008 was estimated to 28.5 and 4.4%, respectively (Zhang et al., 2010). In India, the estimate prevalence of DR reported in clinical examination was 34.1% (Pradeepa and Mohan, 2017).

The pathogenesis of DR is not so far fully understood, although many mechanisms have been proposed such as accumulation of AGEs, increased aldose reductase activity, increased PKC, increased ROS and increased hexosamine flux (Zhang et al., 2012; Safi et al., 2014). Recent evidences has shown that a chronic low level of inflammation also plays key role in pathogenesis of DR (Antonetti et al., 2006; Tang and Kern, 2011). NF-κβ present in sub-retinal membranes and micro-vessels is activated in response to increase ROS and AGEs further activating apoptosis process (Kowluru et al., 2003). The activated NF-κβ further binds to nuclear DNA and over-express different genes leading to production of free radicals and further cell death (Kowluru et al., 2003).

**FIGURE 3:**
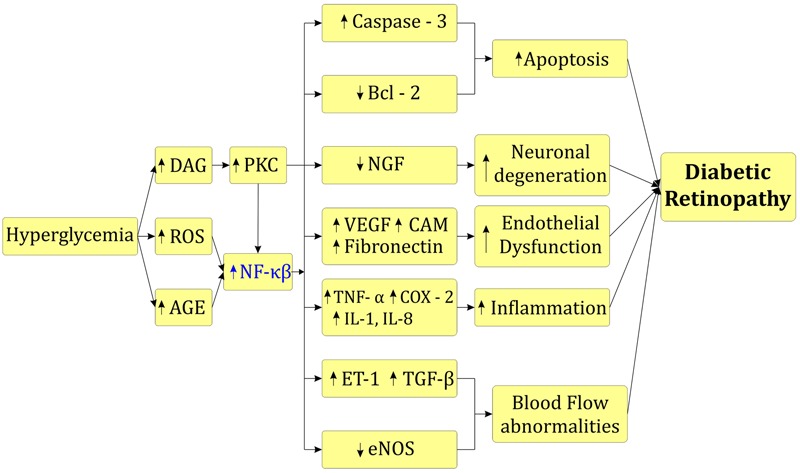
Role of NF-κβ in diabetic retinopathy.

Activated NF-κβ also increases expression of different cytokines such as IL-1β, IL-6, and IL-8 and pro-apoptotic molecule caspase - 3 in vitreous fluid and serum leading to inflammation mediated cell apoptosis (Yuuki et al., 2001; Kowluru and Odenbach, 2004). Increased inflammatory cascade up-regulate ET-1 and down regulate endothelial nitric oxide synthase (eNOS) further leads to narrowing of blood capillaries, retinal ischemia and blood flow abnormalities (De Martin et al., 2000). Up-regulation of VEGF activates NF-κβ that triggers angiogenesis process in diabetic rats (De Martin et al., 2000; Joussen et al., 2001). Activated NF-κβ also over-express intercellular adhesion molecule – 1 (ICAM – 1), fibronectin and CD18 in retinal cells that enhance leukocyte infiltration, retinal fibrosis and blood retinal barrier breakdown (Joussen et al., 2001; Roy et al., 2016). Increased PKC and activated NF-κβ leads to imbalance between proNGF and NGF leading to neuronal dysfunction in the retina (Mysona et al., 2014). Increased polyol flux increase MAPK in renal cells. Furthermore it activates NF-κβ and enhance *trans*-activation of TNF-α and COX-2 leading to inflammation (**Figure 3**; Lorenzi, 2007; Du et al., 2010).

Matrix metalloproteinases (MMPs) also plays an important role in progression of DR. The MMPs, especially MMP-9 is involved in angiogenesis and apoptosis in retinal capillary cells (Kowluru et al., 2012). MMP-9 and MMP-2 are increased in vitreous and retina of diabetic patients and rodents with DR models (Kowluru et al., 2012; Li et al., 2012; Shihab et al., 2015). Activation of NF-κβ, TNF-α and interleukins enhance transcription of MMP-9 leading to DNA alkylation and development of DR (Yan and Boyd, 2007; Kowluru et al., 2016).

## NF-κβ Inhibitors in Diabetic Retinopathy

NF-κβ activation is responsible for inflammation mediated cell apoptosis, fibrosis and angiogenesis (Jiang et al., 2015). The inhibition of NF-κβ related may provide promising alternative treatment for DR. Different natural inhibitors of NF-κβ have been explored for their effect in DR. Treatment with curcumin down-regulated expression of IL-1β, VEGF and NF-κβ and showed significant improvement in DR (Kowluru and Kanwar, 2007).

A clinic based case control study showed that regular Chinese green tea consumption protects DR via inhibition of NF-κβ activation. Epigallocatechin-3- gallate; the tannin abundantly present in Chinese green tea inhibits NF-κβ via inhibition of oxidative stress (Ma et al., 2015). Another medicinal herb *Salvia miltiorrhiza* was explored for its effect in DR. The treatment with *S. miltiorrhiza* inhibited ICAM-1, toll like receptor-4 (TLR-4) and NF-κβ in rats with severe acute pancreatitis (Xiping et al., 2009). *S. miltiorrhiza* also reduced blood sugar, MDA levels and inhibited microaneurysm thereby improved blood retinal barrier in diabetic mice (Zhang et al., 2013). A randomized, double blind, multicenter clinical trial showed that *S. miltiorrhiza* reduced macular edema, neovascularization, venous beading, and cotton spot in patient with DR (Lian et al., 2015). Paeoniflorin, a natural monoterpene glycoside obtained from medicinal plant *P. lactiflora* was tested for its effect in DR in mice. Paeoniflorin ameliorated DR via inhibition of TLR4/NF-κB pathway. It also reduced MMP-9 expression and IL-1β level in retinal cells and vitreous (Zhu et al., 2017).

Resolvin D1 a compound derived from w-3-polyunsaturated fatty acid (PUFA) docosahexaenoic acid (DHA) was studied for its effect in DR. The treatment with resolving D1 showed improvement in retinal matrix accumulation via down-regulation of NF-κβ. Furthermore it also inhibited expression of pro-inflammatory cytokines IL-1β and IL-18 in retina. Resolving D1 also reduced apoptosis in blood retinal barrier via inhibition of NF-κβ mediated activation of caspase-3 and leukocyte infiltration (Yin et al., 2017a). The pyrrolidine dithiocarbamate (PDTC) has been explored for in DR. PDTC inhibited NF-κβ mediated expression of IL-8 and TNF-α and showed significant effect in DR in mice (Yoshida et al., 1999). Astaxanthin, a natural hydroxycarotenoid abundantly present in sockeye salmon, red trout and algae showed protective effect in DR rats. Astaxanthin reduced expression of ICAM-1, and MCP-1 possibly via inhibition of NF-κβ (Yeh et al., 2016). Benfotiamine, an S-acyl derivative of thiamine present in vegetables from allium genus prevented DR through blocking NF-κβ activation by activating transketolase. It also inhibited major three pathways responsible for retinal damage *viz*. PKC pathway, AGEs pathway, and hexosamine pathway (Hammes et al., 2003).

## NF-κβ and Diabetic Cardiomyopathy

Diabetic cardiomyopathy (DC) is the leading cause of mortality in diabetic patients. DC is characterized by systolic and diastolic dysfunction due to reduced contractility, decreased compliance and prolonged relaxation (Patel and Santani, 2009). Increased susceptibility to ischemia/reperfusion injury, accumulation of extracellular matrix and loss of normal micro-vessels are also involved in DC (Bell, 2003; Miki et al., 2013). The number of diabetic patients with cardiovascular complications has been increasing worldwide (Danaei et al., 2011). Some recent studies indicate that the global prevalence of DC in community population is 1.1%. While, 16.9% diabetic patients met with the criteria for DC and 54.4% patients had diastolic dysfunction (Dandamudi et al., 2014).

Prolonged hyperglycemia suppresses glucose oxidation, enhances fatty acid metabolism and modulates intracellular signaling that leads to myocardial injury (Miki et al., 2013). The putative mechanisms of DC include insulin resistance, autonomic dysfunction, and myocardial fibrosis. Hyperglycemia induces oxidative stress, AGE/RAGE and galectin-3 levels, and increases TNF-α in myocardial muscles (Patel and Santani, 2009). Increased AGEs and oxidative stress modulate calcium influx thereby it activates NF-κβ and reduces myocardial contractility (Fang et al., 2004). The increased ROS activates NF-κβ canonically and non-canonically that triggers NF-κβ dependent gene expression and production of pro-inflammatory cytokines IL-6, IL-10 and TNF-α in human heart (Esposito et al., 2002; Jones et al., 2003). The cytokines rapidly degrade IκBα and further activates NF-κβ (Kumar and Sharma, 2010).

**FIGURE 4:**
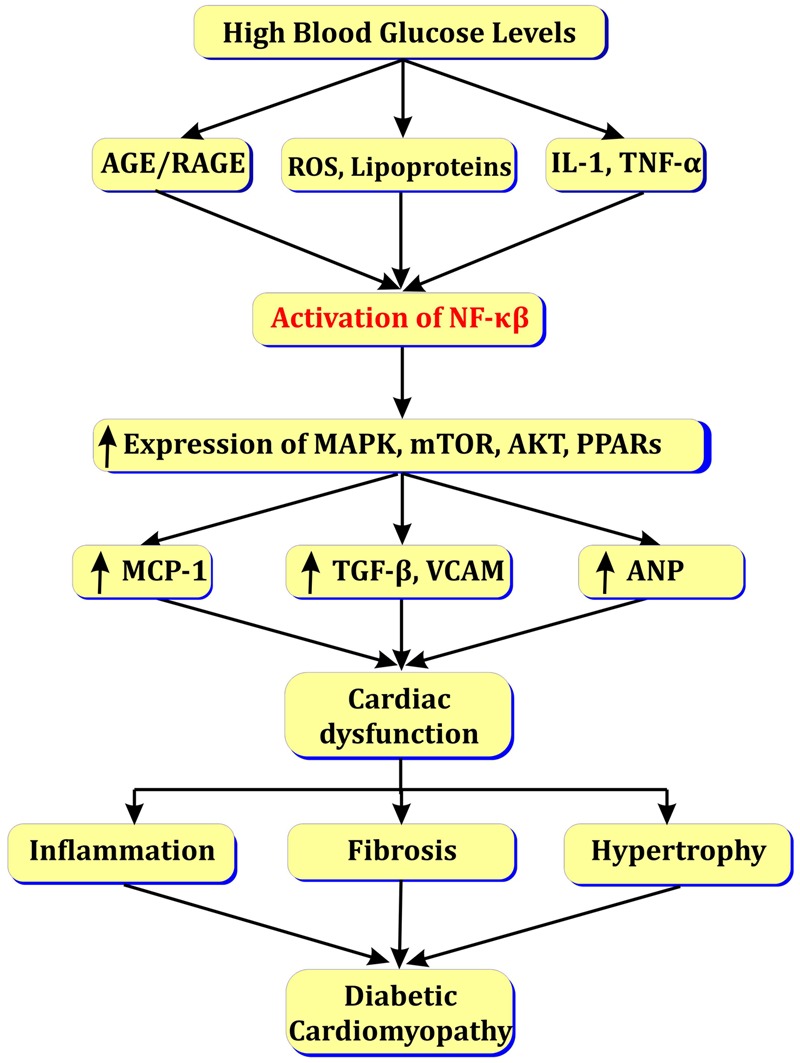
Role of NF-κβ in diabetic cardiomyopathy.

Enhanced fatty acid metabolism in diabetic heart increases levels of LDL/VLDL. Further, LDL and VLDL activate NF-κβ and enhance release of vasoactive amines (angiotensin-II, endothelin-1) and TGF-β that leads to blood flow abnormalities and myocardial fibrosis (Lorenzo et al., 2011). The activation of NF-κβ in myocardial cells may induce myocardial hypertrophy via activation of Toll like receptors (TLRs) (Ha et al., 2005) or by activation of angiotensin-II via MAPK/PPAR pathways (see **Figure 4**; Kawano et al., 2005).

## NF-κβ Inhibitors in Diabetic Cardiomyopathy

The inhibition of NF-κβ may provide effective option in treatment of DC. Recent studies showed that inhibition of NF-κβ inhibits activation of nucleotide-binding oligomerization domain-like receptor protein 3 (NLRP3) and ameliorates DC. NLRP3 inflammasomes takes part in apoptosis process via activation of IL-1β and forming complex with pro-apoptotic molecule caspase-1 and associated speck like protein (ASC). Silencing of NLRP3 leads to inhibition of NF-κβ, activation of IL1β and caspase-1that leads to cardio-protective action (Luo et al., 2014). Very few natural medicines have been explored for their NF-kβ inhibition and cardio-protective activity.

Ginsenoside Rg1, an active component of herbal medicinal plant *Panax ginseng* has been studied for its cardio-protective action in diabetic rats. Ginsenoside Rg1 inhibited myocardial cell apoptosis via inhibition of NF-κβ induced expression of caspase-12 and TNF-α (Yu et al., 2016). The chronic treatment with resveratrol inhibited NF-κβ mediated pro-inflammatory mediators and cell apoptosis. Resveratrol also reduced oxidative stress by increasing catalase and decreasing MDA levels (Mohammadshahi et al., 2014). Hesperetin, an active component of citrus fruit and natural inhibitor of NF-κβ showed cardio-protective activity in diabetic rats. Treatment with hesperetin reduced expression of TNF-α, IL-1β and inhibited myocardial inflammation. It also reduced expression of ICAM-1, VCAM-1 leading to protection form ischemia/reperfusion injury. Additionally it also inhibited expression of collagen I and III leading to reduction in myocardial fibrosis (Yin et al., 2017b). A novel curcumin analog showed C66 [(2E,6E)-2,6-bis(2-(trifluoromethyl) benzylidene) cyclohexanone] showed cardioprotective action via inactivation of NF-κβ. It also inhibited expression of TNF-α and reduced myocardial cell apoptosis (Ren and Sowers, 2014).

## Conclusion

Various preclinical studies have been carried out to study the effect of natural NF-κβ inhibitors in the management of diabetic complications; but its implication in clinical setting is limited. The animal models for diabetic complications depict many clinical features and phenotypes of disease (Chatzigeorgiou et al., 2009). However, no animal model exhibit all features of human diabetic complications. Hence, researchers should consider closely related data such as transcriptomic data, pathological and biochemical data (Betz and Conway, 2016).

NF-κβ is an important player in pathophysiology of vascular complications of diabetes. Inhibition of NF-κβ may provide effective treatment option for diabetic vascular complications. There are numerous natural as well as synthetic NF-κβ inhibitors available but their implications in diabetic complications are very limited. Clinically, NF-κβ is more focused target to overcome resistance chemotherapy (Godwin et al., 2013), management of cancer (Lin et al., 2010), treatment of inflammation (Calzado et al., 2007). Literature suggests that inflammation is one of the part in pathophysiology of diabetic complications. There are limited number of scientific reports with regard to clinical studies of drug molecules in diabetic complications with special focus on NF-κβ as a target. So, there is need to explore potential of NF-κβ inhibitors for their possible effects in diabetic complications with the help of preclinical studies and clinical set up.

## Author Contributions

All authors listed have made a substantial, direct and intellectual contribution to the work, and approved it for publication.

## Conflict of Interest Statement

The authors declare that the research was conducted in the absence of any commercial or financial relationships that could be construed as a potential conflict of interest. The reviewers ED, AT and handling Editor declared their shared affiliation.
